# Sero-prevalence of hepatitis B virus infection and associated factors among health care workers and medical waste handlers in primary hospitals of North-west Ethiopia

**DOI:** 10.1186/s13104-018-3538-8

**Published:** 2018-07-03

**Authors:** Endalew Yizengaw, Tamyalew Getahun, Mekuanint Geta, Wondemagegn Mulu, Mulat Ashagrie, Derese Hailu, Shibabaw Tedila

**Affiliations:** 1 Department of Medical Laboratory Science, College of Medicine and Health Sciences, Bahir Dar University, PO Box 79, Bahir Dar, Ethiopia; 2 Addis Alem Hospital, Bahir Dar, Ethiopia; 3 Department of Medical Microbiology, School of Biomedical and Laboratory Sciences, College of Medicine and Health Sciences, University of Gondar, Gondar, Ethiopia; 4 Amhara Public Health Institute, Bahir Dar, Ethiopia; 5 Department of Human Anatomy, College of Medicine and Health Sciences, Bahir Dar University, Bahir Dar, Ethiopia

**Keywords:** Hepatitis B virus, Health care workers, Medical waste handlers

## Abstract

**Objective:**

The aim of this cross-sectional study was to determined the sero-prevalence of HBV infection and associated factors among health care workers and medical waste handlers in primary hospitals of North-west Ethiopia.

**Results:**

A total of 388 study participants were included in this study. Of which, 268 (69%) were health care workers and 120 (31%) were medical waste handlers. Males accounted 54.9% and the mean age for all study participants was 28.3 (standard deviation = 6.9). Hepatitis B virus surface antigen (HBsAg) was detected in 2.6% health care workers and 2.5% medical waste handlers and the overall hepatitis B virus infection was 10 (2.6%). High rate of hepatitis B virus infection was detected in single participants and those in the age group of 30–40 years were more infected (6.6%). History of contact with HBV infected case (8.3%) (AOR = 6.8, 95% CI = 1.6–28.5, P = 0.009) and history of jaundice (15.4%) (AOR = 10.5, 95% CI = 2.1–12.2, P = 0.03) were statistically associated factors for HBV infection. More than half (54.4%) of the study participants did not take training on infection and 9 (4.3%) of them were positive for HBsAg (COR = 1.3, 95% CI = 0.0.02–1.02, P = 0.052).

## Introduction

Hepatitis is a major public health problem commonly transmitted in health care settings that causes significant mortality and morbidity worldwide [1]. It is mainly caused by Hepatitis viruses, including hepatitis B virus (HBV) [2, 3]. HBV is a DNA virus classified under the family Hepadnaviridae. Humans are the only known natural host for HBV. HBV enters the liver via the bloodstream, and replication occurs in the liver tissue [4]. Globally, 2 billion people are infected with HBV of whom about 360 million are chronic carriers [5–10], 50 million people are newly infected every year [9] and over one million people die each year [10]. The situation is more severe in the developing world particularly in Asia and sub-Saharan Africa [7, 11–13]. Insufficient coverage of HBV vaccination, injection drug users, unsafe blood transfusion, and inadequate health precautions are major risk factors for HBV infection in these regions. [11–13]. World Health Organization (WHO) estimates that the prevalence of HBV infection in Africa is on average more than 10% [12]. Africa has the second largest number of chronic carriers (> 8%) next to Asia [14]. Ethiopia is considered to have medium to high HBV infection rate [15, 16].

Although most infections in the developing world occur in childhood and early adulthood, a significant portion of adults remain at risk [17–24]. Hepatitis B virus infection is a recognized occupational hazard as non-immune health care workers (HCW) and medical waste handlers (MWH) who perform invasive procedures and who handle human specimens are at high risk of exposure [20–25]. Limited vaccination access and poor preventive practices contribute for the high prevalence of HBV infection [26–31]. On top of these, the existing treatment for HBV infection does not provide a complete cure [30, 32]. A protective HBV vaccine has been around since 1982 [30, 31, 33–35]. But HCWs and MWHs in the developing world have poor access to this vaccine [35–39].

Even though few studies had indicated intermediate to high HBV infection in Ethiopia, no sufficient data among HCWs and MWHs. On the other hand, HCWs and MWHs are at high risk of transmitting to the community. This study showed the sero-prevalence of HBV and associated factors among HCWs and MWHs. Besides, this work is used as a benchmark for policy makers and stakeholders. It is also important beyond the country as it adds in the existing knowledge in HBV infection and associated factors.

## Main text

### Methods and materials

#### Study design and period

A hospital based cross-sectional study was conducted from September 2017 to December 2017. HCWs and MWHs were included from different primary hospitals. The number of HCWs was allocated proportionally to each hospital. Random sampling technique was employed to select HCWs at each hospital and all MWHs were included in the study.

#### Study area and setting

This study was conducted at primary government sponsored 50 bed hospitals around Bahir Dar, North-west Ethiopia. These hospitals serve about one million people. Bahir Dar is the capital city of Amhara National Regional State (ANRS), Ethiopia. It is 570 km away from Addis Ababa, the capital, in the North-west direction and situated in the south of Lake Tana, the biggest lake in the country. The altitude ranges from 1799 m to 5902 ft above sea level and the coordinates are 11°35.6184′ N latitude and 37°23.4462′ E longitude. There are different HCWs, MWHs and supporting staffs in each hospital. Of whom, HCWs and MWHs have high risk of exposure for various infectious agents like HBV and high possibility of transmitting the infection to clients.

#### Data collection

Self-administered structured questionnaire was used to collect socio-demographic characteristics of the study participants and associated risk factors.

#### Laboratory procedure

##### Sample collection

Five milliliters of venous blood were collected from each study participant by plane tube (BD Vacutainer) following standard operating procedure for collection of blood. Blood was centrifuged at 300 RPM for at least 10 min at room temperature and the serum obtained was collected with eppendorf tube. Collected serum was store at − 20 °C if not processed immediately. The serum sample was used to detect hepatitis B surface antigen.

#### Detection of hepatitis B virus surface antigen (HBsAg)

Tests for the HBV infection can be made by demonstration in sera of specific markers of infection. Screening of hepatitis B surface antigen (HBsAg) was performed for diagnosis of HBV infection in this study. From each collected serum sample HBsAg was tested using an enzyme-linked immuno sorbent assay (ELISA), Hepanostika ELISA kits (Hepanostika HBsAg Uni-Form II, bio-Mérieux, Boxtel, The Netherlands) following the manufacturer protocols.

#### Quality control

Blood sample was collected by experienced laboratory practitioners. All the reagents were checked for expiry date, appropriate storage temperature and humidity before use. Positive and negative controls were run together with each sample testing. The ELISA washer was rinsed before every washing procedures and ELISA reader was calibrated before running the test. The detection of HBsAg was done strictly following the manufacturer’s instruction. Collected data was checked for completeness and consistencies.

#### Statistical analysis

Data were entered and analyzed using Statistical Package for Social Science 22 (IBM Corp- Released 2011.IBM SPSS statistics Armonk, NY: IBM Corp). Descriptive statistics was used to describe the demographic characteristics of participants with HBV infection. Bi-variable logistic analysis was used to identify factors associated with HBV infection. A multivariable logistic regression was used to assess the impact of the independent variables on the outcome variable. Odds ratios (OR) was used as a measure of the strength of association and P value ≤ 0.05 was considered statistically significant.

### Results

#### Socio-demographic data

A total of 268 HCWs (69%) and 120 MWHs (31%) were participated in this study. Males accounted 54.9% and the mean age for all study participants was 28.3 (standard deviation = 6.9) and the minimum and maximum age was 19 and 54 years respectively. Majority (94.8%) of the participants were orthodox Christians. Regarding the marital status of participants about 49% were single and 45.1% were married. Among HCWs participated in the study, 45.9% were nurses (including midwifes) and 9.3% were doctors (including emergency surgeries). Laboratory practitioners and pharmacists accounted 8 and 5.9% respectively (Table 1).

**Table 1:** Socio-demographic characteristic of health care workers and medical waste handlers in primary hospitals around of North-west Ethiopia, 2018

Variables	Frequencies	Percentage (%)
Age (years)		
21–29	280	72
30–40	76	19.6
41–50	29	7.5
+50	3	8
Gender		
Male	175	54.9
Female	213	45.1
Religion		
Orthodox	368	94.8
Muslim	18	4.6
Others	2	0.5
Marital status		
Single	190	49
Married	175	45.1
Divorced	5	1.3
Widowed	6	1.5
Separated	12	3.1
Occupation		
Nurse	178	45.9
Doctor	36	9.3
Laboratory	31	8
Pharmacy	23	5.9
Medical waste handler	120	30.9

#### Sero-prevalence of hepatitis B virus infection

From the total 268 HCWs and 120 MWHs screened for hepatitis B surface antigen, 7 (2.6%) and 3 (2.5%) were positive respectively and the overall hepatitis B virus infection was 10 (2.6%). High rate of hepatitis B virus infection was detected in single participants and those in the age group of 30–40 years were more infected (6.6%). Increased proportion of HBV was detected in males (OR = 1.2, 95% CI = 0.35–4.3, P = 0.75), Age group 30–40 (OR = 1.1, 95% CI = 0.10–12.7, P = 0.93) but it was not statistically significant (Table 2).

**Table 2:** Demographic characteristics and hepatitis B virus infection among health care workers and medical waste handlers in primary hospitals around of North-west Ethiopia, 2018

Variables	N (%)	HBsAg (N = 388)		
		n (%)	COR (95% CI)	P value
Sex				
Male	175 (45.1)	5 (2.9)	1.2 (0.35–4.3)	0.75
Female	213 (54.9)	5 (2.3)	1	
Age				
19–29	280 (72.2)	7 (2.5)	1.2 (0.15–10)	0.85
30–40	76 (19.6)	2 (2.7)	1.1 (0.10–12.7)	0.93
≥ 41	32 (8.2)	1 (3.0)	1	
Marital status				
Single	190 (49.0)	6 (3.2)	1	
Married	175 (45.1)	3 (1.7)	1.9 (0.46–7.6)	0.38
Divorced	23 (5.9)	1 (4.3)	0.72 (0.08–6.2)	0.76
Profession				
Nurse + Doctor	214 (55.2)	4 (1.9)	1	
Laboratory	31(8)	2 (6.5)	0.23 (0.05–1.6)	0.15
Pharmacy	23 (5.9)	1 (4.3)	0.42 (0.04–3.9)	0.45
Medical waste handler	120 (30.9)	3 (2.5)	0.74 (0.16–3.4)	0.70

#### Associated factors for hepatitis B virus infection

The following associated factors [11–13] were assessed for hepatitis B virus infection: history of blood transfusion, history of circumcision, history of jaundice, history of needlestick injury vaccination status, history of hospitalization, history of unprotected sex, history of previous dental and surgical procedures, history of contact history and training status were some of the factors. Of which, history of jaundice and history of contact with HBV infected case had independent and statistically significant association with multivariable logistic regression. It was showed that 26 (6.7%) and 80 (20.6) study participants had a history of jaundice and multi-sex partner respectively. Of the total study participants only 36 (9.3%) were vaccinated for HBV and 60 (15.5%) had history of contact with HBV infected case. There were 24 (6.2%) study participants who had history of sharing dental materials during dental procedures. Among those who had a history of contact with HBV infected case, 5 (8.3%) were positive for HBsAg and this was statistically significant (AOR = 6.8, 95% CI = 1.6–28.5, P = 0.009). Three (12.5%) of participants with history of sharing dental materials during dental procedures were positive for HBsAg (AOR = 4.0, 95% CI, 0.81–20.4, P = 0.09). Among those who had history of jaundice, four (15.4%) were positive for HBsAg (AOR = 10.5, 95% CI = 2.1–12.2, P = 0.004) and 5 (6.2%) of the study participants who had multi-sex partner were positive for HBsAg (AOR = 1.5, 95% CI = 0.33–6.6, P = 0.60).

More than half (54.4%) of the study participants did not take training on infection prevention protocols of various infectious diseases including HBV and 9 (4.3%) of them were positive for HBsAg (COR = 1.3, 95% CI = 0.0.02–1.02, P = 0.052) (Table 3).

**Table 3:** Hepatitis B virus infection and associated factors among health care workers and medical waste handlers in primary hospitals around of North-west Ethiopia, 2018

Variables		N (%)	HBsAg positive n (%)	COR (95% CI)	P value	AOR (95% CI)	P value
Transfusion	Yes	128 (33)	4 (3.1)	1.4 (0.38–4.9)	0.63		
	No	260 (67)	6 (2.3)	1			
Circumcision	Yes	256 (66)	8 (3.1)	2.1 (0.44–10)	0.53		
	No	132 (34)	2 (1.5)	1			
Jaundice	Yes	26 (6.7)	4 (15.4)	10.8 (2.8–41)	<0.001	10.5 (2.1–12.2)	0.004
	No	362 (93.3)	6 (1.7)	1			
Needlestick injury	Yes	72 (18.6)	2 (2.8)	1.1 (0.23–5.3)	0.90		
	No	316 (81.4)	8 (2.5)	1			
Lack of vaccination	Yes	36 (9.3)	1 (2.8)	1			
	No	352 (90.7)	9 (2.6)	1.1 (0.13–8.8)	0.94		
Hospitalization	Yes	123 (31.7)	5 (4.1)	2.2 (0.63–7.7)	0.22		
	No	265 (68.3)	5 (1.9)	1			
Surgical procedures	Yes	64 (15.5)	2 (3.1)	1.3 (0.26–6.1)	0.63		
	No	324 (84.5)	8 (2.5)	1			
Unprotected sex	Yes	103 (26.5)	5 (4.9)	2.8 (0.8–10)	0.10		
	No	285 (73.5)	5 (1.8)	1			
Multi-sex partner	Yes	80 (20.6)	5 (6.2)	4 (1.1–14)	0.03	1.5 (0.33–6.6)	0.60
	No	308 (79.4)	5 (1.6)	1			
Dental procedures	Yes	24 (6.2)	3 (12.5)	7.3 (1.7–30)	0.006	4.0 (0.81–20.4)	0.09
	No	364 (93.8)	7 (1.9)	1			
Contact history	Yes	60 (15.5)	5 (8.3)	5.9 (1.6–20.9)	0.006	6.8 (1.6–28.5)	0.009
	No	328 (84.5)	5 (1.5)	1			
Training	Yes	177 (45.6)	1 (0.6)	1			
	No	211 (54.4)	9 (4.3)	1.3 (0.02–1.02)	0.052		

### Discussion

This study showed the prevalence of HBV infection among HCWs and MWHs at primary hospitals. Though HBV infection is preventable and there is a protective vaccine, it remains a public health problem world-wide especially in Africa where Ethiopia is no exception. HCW and MWH are among high risk groups for infectious disease including HBV. Estimates of HBV infection among HCW and MWH are needed to know the disease burden and thus to establish protective strategies. Yet limited data is available on the sero-prevalence of HBV infection in this risk groups in Ethiopia. Hepatitis B virus infection can be diagnosed by detecting different markers associated with the virus and/or with the immune response. In the present study we use HBsAg as a marker of infection to diagnose HBV infection.

The present study had shown that HBsAg was detected in 2.6% of HCWs and 2.5% MWHs with overall prevalence of 2.6%. The sero-prevalence of HBV in HCWs in this study was higher than the study conducted in tertiary hospital of Indian HCW (0.4%). This difference could be due to the low vaccination coverage and shortage of training on infection prevention in Ethiopia. In this study we found that only 36 (9.3%) of the HCW and MWH were vaccinated against hepatitis B virus. This is much lower than the report in India where 50% of HCW were fully vaccinated and the WHO hepatitis B vaccine coverage estimate in African HCW where the lowest is 18% [12, 38]. The detection of HBsAg among MWHs in the present study was lower than the report from southern Ethiopia (1.3%) where 7.2% of participants were vaccinated against HBV [39].

However, the detection rate of HBsAg in MHWs and HCWs was lower than previous studies conducted in Addis Ababa (6.3%) and Gondar (6.0%) [40, 41] and in Sudan (4.4%), Tanzania (7.4%) and Uganda (8.1%) [42–44] respectively. Only 20% of HCWs in Tanzania were vaccinated and none of the MWHs in Gondar and HCWs in Sudan was vaccinated for HBV and there might be improvements in awareness on safety procedures and the importance of vaccination.

The occupational risk of HBV infection in this study was high for the laboratory practitioners and MWHs. This indicates that there is a difference in level of risk of exposure for HBV infection among different professions. Similar result had been reported from Uganda [43, 44]. Still there is no regular and programmed protective vaccination program against HBV in Ethiopia for high risk groups like HCWs and MWHs. Indeed this study show that individuals with no training in infection prevention had a higher risk of exposure (though not significant) to hepatitis B virus infection [43, 44]. In this study, a statistically significant association was observed between HBV infection and history of jaundice (P = 0.004) and history of contact with HBV positive cases (P = 0.009). This signifies that jaundice can be considered as a suggestive indicator for HBV infection so that further diagnosis with specific tests is required to rule out other infections and health conditions.

Though there is no concert national prevalence data [45] to discuss with, the detection rate of HBsAg in MHWs and HCWs in this study was lower than reports from population based studies in the country. A five decade (1968–2015) systematic and meta-analysis study conducted in Ethiopia showed a pooled prevalence of 7.4% [45]. In the same study, higher prevalence of HBsAg is reported among HCW (7.3–9.0%) and medical MWH (6.0–6.3%). A community based cross-sectional study showed 3.1% HBV prevalence by detecting HBsAg [46]. This might be due to the decline in the prevalence of HBV infection in the country. The relative improvement in the accessibility of the protective vaccine and awareness regarding the prevention of the disease might contribute in the reduction of the HBV infection. On the other hand it might indicate that working in the hospital seething might be protective against HBV. It is plausible that those who are working in the hospital get trainings on the prevention of infectious disease more frequent than the community. They are also given a priority in vaccination as they are considered as risk groups since they deal with sort of infected sample. Most importantly the level of education and their field of specialty, especially for health care workers, are also better than the community [45, 46] so that they have expected to have better awareness regarding the precautions and safety of blood borne transmission in general and HBV infection prevention in particular.

### Conclusion

The occupational risk of HBV infection among the HCWs and MWHs in this study was high. HBV vaccine and trainings on infection prevention should be more readily available for these high risk groups.

## Limitations of the study

This study didn’t determine other potential infectious pathogens in health care settings like hepatitis C virus and HIV.

## Abbreviations

DNA: deoxyribonucleic acid
ELISA: enzyme linked immuno sorbent assay
HBsAg: hepatitis B surface antigen
HBV: hepatitis B virus
WHO: World Health Organization
